# Polarization dependent photocurrent in the Bi_2_Te_3_ topological insulator film for multifunctional photodetection

**DOI:** 10.1038/srep14184

**Published:** 2015-09-16

**Authors:** J. D. Yao, J. M. Shao, S. W. Li, D. H. Bao, G. W. Yang

**Affiliations:** 1 State Key Laboratory of Optoelectronic Materials and Technologies, Nanotechnology Research Center, School of Physics & Engineering, Sun Yat-sen University, Guangzhou 510275, Guangdong, P. R. China

## Abstract

Three dimensional Z_2_ Topological insulator (TI) is an unconventional phase of quantum matter possessing insulating bulk state as well as time-reversal symmetry-protected Dirac-like surface state, which is demonstrated by extensive experiments based on surface sensitive detection techniques. This intriguing gapless surface state is theoretically predicted to exhibit many exotic phenomena when interacting with light, and some of them have been observed. Herein, we report the first experimental observation of novel polarization dependent photocurrent of photodetectors based on the TI Bi_2_Te_3_ film under irradiation of linearly polarized light. This photocurrent is linearly dependent on both the light intensity and the applied bias voltage. To pursue the physical origin of the polarization dependent photocurrent, we establish the basic TI surface state model to treat the light irradiation as a perturbation, and we adopt the Boltzmann equation to calculate the photocurrent. It turns out that the theoretical results are in nice qualitative agreement with the experiment. These findings show that the polycrystalline TI Bi_2_Te_3_ film working as a multifunctional photodetector can not only detect the light intensity, but also measure the polarization state of the incident light, which is remarkably different from conventional photodetectors that usually only detect the light intensity.

Topological insulator (TI) is an unconventional phase of quantum matter possessing insulating bulk state as well as time-reversal symmetry protected Dirac-like surface state^1^,^2^,^3^,^4^,^5^,^6^,^7^,^8^,^9^,^10^. Such spin-momentum lock surface states have been extensively investigated by surface sensitive detection techniques including angle-resolved photoemission spectroscopy (ARPES)^1^,^2^,^3^,^4^ and scanning tunneling microscopy (STM)^5^,^6^,^7^. In the past few years, photonic and optoelectronic device based on such unique helical surface states have attracted plenty of interests. Large amount of theoretical efforts have focused on the response of the surface states to light. And many potential exotic phenomena are found, including surface state assisted high-performance broadband photodetection^8^, circularly polarized light induced helicity-dependent direct current^9^ and gapped Dirac cone^10^, as well as warping effect induced optical absorbance increment^11^. Excitingly, polarized light controlled photocurrents^12^, photo-dressed Floquet-Bloch states^13^ and topological surface state enhanced photothermoelectric effect^14^ are subsequently observed on the surface of TI films. Additionally, Olbrich *et al.* reported the anisotropy photogalvanic effects induced by asymmetric scattering of Dirac fermions driven back and forth by the terahertz electric field^15^. Although significant progresses have been made in the study of interaction between TIs and light, most of these studies are based on more costly single crystal TI^12^,^13^,^14^,^15^. There have been no reports involved in light assisted-electrical transport characteristic, especially the dc photocurrent response of TI film under linear polarized light irradiation, so far. In fact, high-quality polycrystalline Bi_2_(Te, Se)_3_ films^16^,^17^,^18^ prepared by pulsed-laser deposition (PLD) have demonstrated to be an attractive material platform for exploring TI’s intrinsic nature and real application, such as the far-infrared photodetection^19^, magnetoresistance switch effect^20^, Josephson supercurrent^21^ and weak antilocalization^22^. To exploit photodetector applications of the TI film, we have thus carried out a study of the light assisted-electrical transport under the linear polarized light irradiation. Here, we report the first observation of novel polarization dependent photocurrent of the polycrystalline Bi_2_Te_3_ film under the linear polarized light based on the mode of photoresistor-type photodetector. The photocurrent response exhibits linearly dependent on both the light intensity and the bias voltage. To address this polarization dependent photocurrent, we use a simple massless Dirac fermion surface state model, and adopt the perturbation theory and Boltzmann equation to treat the light irradiation and the dc electric field caused by the bias voltage, respectively. Importantly, the theoretical surface photocurrent shows a nice qualitative agreement with the experiments.

## Results

**Figure 1:**
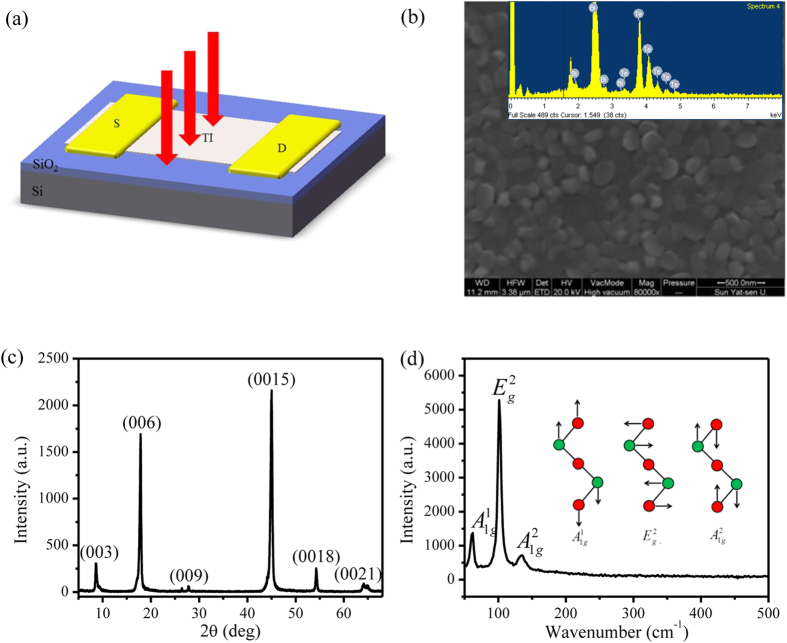
Device fabrication and characterization. (**a**) Schematic diagrams of the device structure and photocurrent measurement setup in a two pole structure. (**b**) SEM image of the PLD-growth polycrystalline Bi_2_Te_3_ film, scale bar: 500 nm. Inset: Energy dispersive spectroscopy. (**c**) Raman spectrum with the 514 nm excitation laser. The three characteristic peaks represent 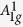 (60.3 cm^−1^), 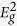 (101.3 cm^−1^) and 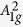 (133.1 cm^−1^) mode, respectively. The side views of three vibration modes are shown in the inset. (d) XRD diffraction pattern. It only shows the (003) family diffraction peaks of Bi_2_Te_3_. All the characterizations suggest that the PLD Bi_2_Te_3_ film is of high crystalline quality.

The polycrystalline Bi_2_Te_3_ films, 2 mm in length (distance between electrodes), 100 m in width and 200 nm in thickness, are prepared by PLD with the help of a shadow mask and the detailed preparation process have been described elsewhere^16^. The schematic diagram of the device is shown in Fig. 1(a). Note that 300 nm SiO_2_ layer was firstly prepared on the Si substrate. The Bi_2_Te_3_ film is deposited onto the inner portion of the substrate. Pt electrodes of 100 nm in thickness are deposited onto the two ends of the Bi_2_Te_3_ film by an ion sputtering technique. Such configurations can effectively prevent unintentional contact between the Pt electrodes and the underneath Si through the margin of the substrate and avoid the interference of the underneath Si channel on the transport measurement of the TI Bi_2_Te_3_ film. I-V characteristic measurement is performed (Supplementary Section S1). And the linear I-V curve indicates the Ohmic contact between the Pt electrodes and the Bi_2_Te_3_ film, which further ensures that the observed response can only come from the Bi_2_Te_3_ film itself instead of the common Schottky junction between the sample and the electrodes. The electrical characteristic of the Bi_2_Te_3_ film is evaluated using a Keithley 4200-SCS semiconductor parameter analyzer. All measurements were performed at room temperature under ambient condition. Details of the measurement setup are described in Supplementary Section S2.

Scanning electron microscope (SEM), Energy Dispersive Spectrometer (EDS), Raman spectroscopy and X-ray diffraction (XRD) were used to study the morphology, constituent and structure of the PLD-growth Bi_2_Te_3_ film. Figure 1(b) presents a typical SEM image. Large scale uniform surface morphology with arbitrary oriented hexagonal crystal grain can be seen. The EDS analysis in the inset shows that the film possesses a stoichiometry atomic composition ratio of Bi to Te approaching 2:3. Representative Raman spectroscopy with the 514 nm excitation laser is shown in Fig. 1(c). The three peaks corresponds to the 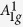, 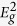 and 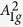 vibration mode, as shown in the inset of Fig. 1(c). The X-ray diffraction pattern in Fig. 1(d) only possesses the (003) family diffraction peaks, indicating that the sample is highly c-axis oriented. All characterizations above reveal that the PLD-growth Bi_2_Te_3_ film is of high-crystalline quality and can be used to investigate the intrinsic properties of TI.

**Figure 2:**
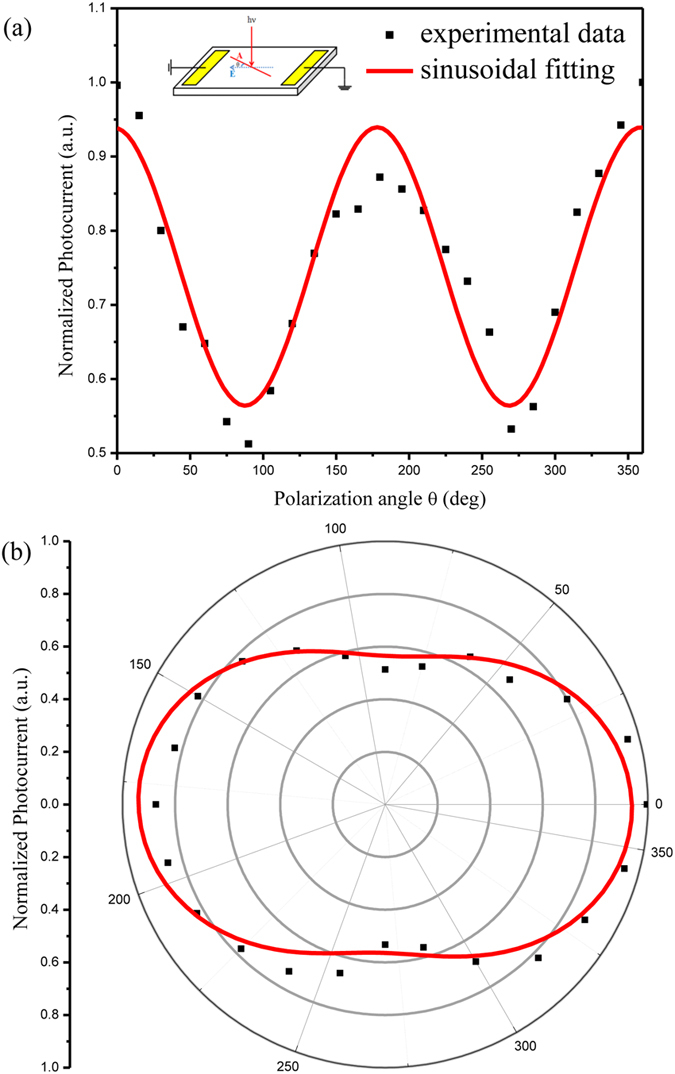
Polarization dependent photocurrent of the TI Bi_2_Te_3_ film (a) in Cartesian coordinate, and (b) in polar coordinates. The wavelength of light is 635 nm, the bias voltage is 1 V and the energy density of the light is 35 mW/cm^2^. The black dots are the experimental data and the red lines are the sinusoidal function fittings. The insert in (**a**) is schematic for the photocurrent transport measurement, where **A** is the light’s vector potential, indicating the polarization direction, **E**_0_ is the dc electric field caused by the bias voltage, and *θ* is the angle between them.

The photocurrent of the Bi_2_Te_3_ film under 635 nm linearly polarized light is studied under configuration shown in the inset of Fig. 2(a). The observed photocurrent shows the distinct polarization dependence. In detail, the photocurrent exhibits oscillation with a periodicity of 180° as the normally incident light’s polarization angle is rotated. Red lines are fits to a sinusoidal function. In order to have a more intuitive perspective of the nature of the photocurrent, the data is also shown in polar coordinates in Fig. 2 (b), which exhibits obvious anisotropy behavior. When the light is polarized along the direction of the bias voltage, the photocurrent reaches the maximum value. As the light polarization direction changes from parallel to perpendicular to the direction of the bias voltage, the photocurrent decreases systematically. When the light is polarized perpendicular to the direction of the bias voltage, the photocurrent reaches the minimum value. The ratio of the maximum to minimum photocurrent is 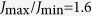. To exclude the possibility that the observed polarization dependence photocurrent might originate from some unintentional artifacts of the experimental set up, the angle dependent power of the polarized light was measured. It exhibited the isotropy behavior (Supplementary Section S1). Besides, we rotate the sample by 90° and repeat the measurements under the identical measurement configuration. The results are summarized in Supplementary Section S3. Expectedly, almost the same results are obtained. Therefore, it can be unambiguously claimed that we realize the first experimental demonstration of the dc polarization dependent photocurrent in the TI Bi_2_Te_3_ film.

**Figure 3:**
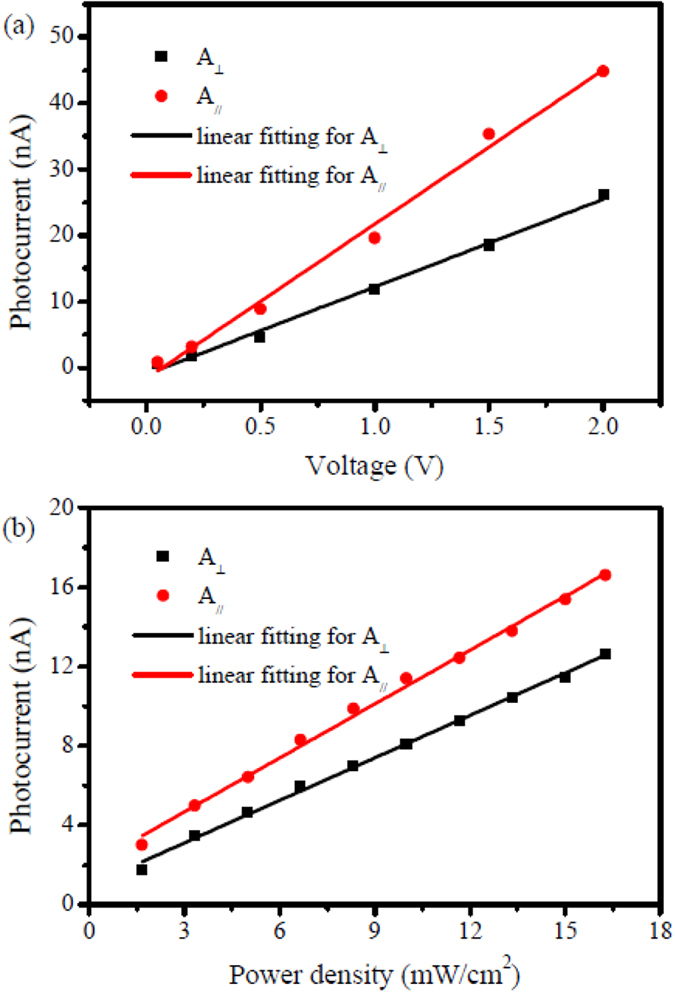
(**a**) Voltage-dependent and (**b**) power-dependent photocurrent under the linearly polarized light. 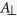 and 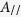 refer to lights whose polarization direction isperpendicular and parallel to the bias voltage, respectively. The dots are experimental data and the solid lines are linear fittings.

To have a further insight into the properties of the polarization dependent photocurrent of the Bi_2_Te_3_ film, the influence of the bias voltage and the incident light intensity on the photocurrent was investigated. Figure 3(a) presents the voltage dependent photocurrent under the linear polarized light irradiation. In general, the photocurrent with the light polarization both parallel and perpendicular to the direction of the bias voltage is linearly dependent on the voltage bias. In fact, stronger electric field across the channel may separate and collect the photo-generated carriers more efficiently. And the linear photocurrent-bias voltage relation indicates a constant photoconductivity that doesn’t change with the applied electric field. It is quite reasonable as the bias voltage applied here is sufficiently low so the linear response mainly takes control. Figure 3(b) presents the power dependent photocurrent under the polarized light irradiation. The photocurrents with the light polarization both parallel and perpendicular to the direction of the bias voltage also increase linearly with the increase of the light’s excitation power density which is proportional to the light intensity. As we know, light with higher intensity is supposed to induce more photo-generated carriers. Under some certain circumstances, like weak light irradiation and uniform life time (*i.e.* homogeneous recombination) for the photo-generated carriers, the linear photocurrent response to light intensity is important, which we will give a detail theoretical explanation later.

Next, we seek for the possible origin of the polarization dependent photocurrent in the TI Bi_2_Te_3_ film above. One possible reason for the anisotropic property in photocurrent is the crystallographic anisotropy. Typically, the crystallographic anisotropy would lead to a strong anisotropy in electronic band structure and finally result in the anisotropy response to the polarized irradiation^23^,^24^. Very recently, Olbrich *et al.* pointed out that crystallographic can lead to asymmetric scattering for carries in TIs under a terahertz electric field without bias voltage^17^. Therefore, anisotropy photocurrent analogous to what we observed generated^15^. However, the asymmetric-scattering effect is rather weak. It is easily submerged when the bias voltage is applied to the sample. Besides, the PLD growth Bi_2_Te_3_ film possesses the rhombohedral structure and is highly c-axis oriented as conformed by the SEM image and XRD pattern. Thought the film is anisotropy between the c axis and any direction in the plane of the film, it is isotropic in the plane of the film due to its polycrystal nature, and arbitrary orientation of crystal grain in the plane of the film has been shown before in Fig. 1(b). Therefore, crystallographic anisotropy could not well explain the anisotropic photocurrent here. Another possible reason is the mixing of valence bands due to quantum confinement^25^,^26^,^27^ or electric field attenuation of perpendicular polarization light compared to parallel polarization light originated from large dielectric contrast^28^. Yet they can occur only when the dimension of the sample is much smaller than the wavelength of the incident light. In contrast, the size of our sample is orders of magnitude larger than that of the wavelength of the incident light in any direction in the film plane. Therefore, this also does not seem to be a reasonable explanation.

## Discussion

Eventually, we attribute the polarization dependent photocurrent in our sample to the helical surface state of the TI film, similar to what is found in graphene^29^,^30^. When the sample is under the light irradiation, the electrons in the valence band will absorb photons and be excited to the conduction band. And the transition probability can be obtained from the Fermi’s golden rule 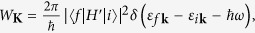 where 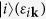 and 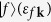 indicate the initial and final state (energy), respectively. Through Peierl’s substitution, the electron-photon interaction is 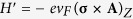 if we consider the simple original Hamiltonian of our topological insulator sample’s surface state is 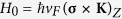. **A** is the vector potential of the normally incident linearly polarized light used in our experiment, and it can be expressed as 
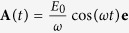
, 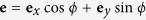 with *ϕ* displaying the light’s polarization angle, and *E*_0_ is the electric field amplitude related to the average.

It is to be note that the Bi_2_Te_3_ film in our experiment is polycrystalline instead of single crystalline. Therefore, one will doubt if the original Hamiltonian for single crystal TI is suitable to describe the polycrystalline TI samples in our experiment. We suspect the answer is yes for the following reasons. Firstly, SDH oscillations as well as Josphson supercurrent were demonstrated in polycrystalline Bi_2_Te_3_ samples synthesised by the Czochralski method^21^. Besides, metallic surface states as well as the weak antilocalization (WAL) effect have been demonstrated in the PLD-grown Bi_2_Te_3_ films in our previous works and those of other groups^16^,^17^,^22^,^31^. These works have demonstrated the existence of time-reversal symmetry-protected topological surface states in the polycrystalline Bi_2_Te_3_ films, i.e. the polycrystalline Bi_2_Te_3_ film provides an reliable material platform for exploring the TI’s intrinsic nature. Most importantly, as demonstrated in our previous work, the PLD-grown polycrystalline Bi_2_Te_3_ film is consist of many c-axis oriented Bi_2_Te_3_ grains^16^. Every grain is a small single crystal TI. For each grain, the angle dependent photocurrent can be well deduced by the standard Dirac equation of single crystal TI. For a total polycrystalline film, the photoresponse is suspected to be the sum of all grains, which is reasonable because the polycrystalline film is highly c-axis oriented and isotropy for carrier scattering in all directions in the plain. Consequently, the angle dependent photocurrent of a polycrystalline film is still in qualitatively agreement with that of the single crystal sample. That is, the polycrystalline topological surface states can still be qualitatively described by the Dirac equation of the single crystal TI.

Then, following the above calculation and we have


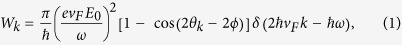


It indicates the transition probabilities heterogeneous in the momentum space under linearly polarized light illumination. The incident linearly polarized light preferentially excites electrons whose momentum is perpendicular to the light’s polarization.

We introduce a lifetime *τ*_*i*_ of the generated photoelectrons to include all the recombination effect under continuous light irradiation. Then the steady state electron distribution function can be obtained by balancing the carriers’ generation rate and its relaxation rate.


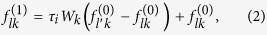


where 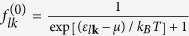 is the Fermi-Dirac distribution function with chemical potential *μ* at temperature *T*.

Then applying a spatially-homogeneous dc electric filed **E**_0_ to the sample’s surface state, we can have its non-equilibrium distribution function 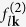 in steady state from the Boltzmann kinetic equation within momentum relation time approximation


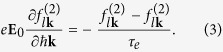


Here, 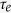 is the momentum elastic relaxation time which is mainly controlled by the impurity scattering. In our polycrystal topological insulator sample, the distribution of disorder and impurity is uniform, so *τ*_*e*_ is assumed to be momentum independent and isotropy.

As our experimental photocurrent shows a good linear relation with the applied electric voltage, we solve 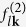 to the first order of electric field **E**_**0**_. Then, with electron’s velocity 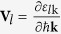, the current density of the sample can be expressed as


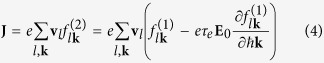


So we have the photocurrent


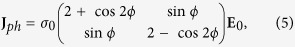


with 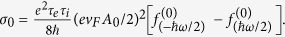


**Figure 4:**
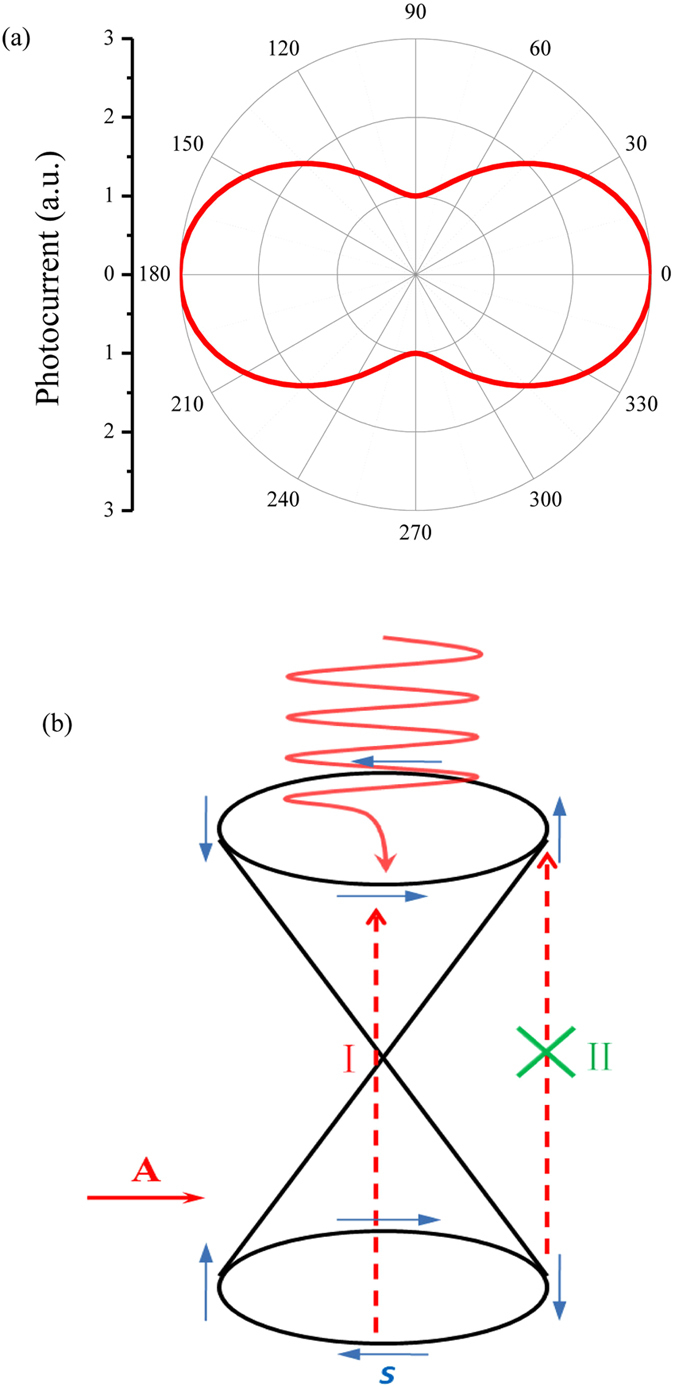
(**a**) Theoretical calculated longitudinal photocurrent’s dependence on the polarization of light, *θ* is shown in the insert of Fig. 2(a). (**b**) Schematically illustration of the selective excitation under linear polarized light. Blue arrows indicate the spin directions of the electrons. A is the incident light’s vector potential and s is the electron’s spin.

From Eq. (5), we know that the photocurrent is proportional to the intensity of light with certain frequency, which is consistent with our experimental results. Besides, the photoconductivity tensor *σ*_*ph*_ shows a distinct anisotropic phenomenon, and reveals that the photocurrent is polarization dependent. The maximum photocurrent is obtained when the light is polarized along the electric field, and it is three times larger than the minimum one when the light is polarized perpendicular to the electric field (show in Fig. 4(a)). And the polarization dependent longitudinal photocurrent matches our experimental results quite well except the ratio of 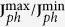, which also makes sense because we only consider a very simple surface state Hamiltonian here and exclude all the other complicated modifications such as the unavoidable warping of the surface states and doping of the sample. Another reason for the deviation of the experimental results from the theoretical calculation is the effect of the bulk contribution. An isotropic portion of photocurrent from the bulk may weaken the degree of polarization caused by the surface state. This is beyond the capacity of our experimental conditions and the scope of this article.

Actually, the above anisotropic photocurrent is due to the spin selective rule-induced heterogeneous electron excitation in momentum space when the sample is under linearly polarized light illumination, seen from transition probability in Eq. (1). To have a more intuitive physical perspective, the excitation mechanism is schematically depicted in Fig. 4(b). In brief, if the light is polarized along the spins of the excited particles, the electron-hole excitation is maximal (Step I in Fig. 4(b)). On the contrary, if the light is polarized perpendicular to the spins of the excited particles, the excitation is forbidden (Step II in Fig. 4(b)). Consequently, incident lights with different polarization states excite electrons with different spins, thus leading to different non-equilibrium distribution in steady state under a fixed external electric field and resulting in different photocurrent. Since the surface state of TI is spin-momentum locked, the resulting photocurrent depends on the angle between **A** and **J**.

In summary, we have demonstrated the anisotropic photocurrent for photodetectors based on the polycrystalline TI Bi_2_Te_3_ film under the polarized light irradiation under the bias voltage condition. It was found that the response photocurrent is linearly dependent on both the bias voltage and the light intensity. Meanwhile, we have established a theoretical treatment based on perturbation theory and Boltzmann equation to pursue the anisotropic photocurrent in TI films and the theoretical results were in nice qualitative agreement with the experimental data. These findings open a door to applications of polycrystalline TI films as a multifunctional photodetector which can not only detect the light intensity, but also measure the polarization state of the incident light, which is remarkably different from conventional photodetectors that usually only detect the light intensity.

## Method

We use pulsed-laser deposition (PLD) to prepare polycrystalline TI Bi_2_Te_3_ thin films^18^. In our experiments, the deposition parameters are as follows. The base pressure of the growth chamber is better than 1 × 10^−4^ Pa. The substrates used in the PLD-growth are (100) oriented single crystal Si wafers with a 100 nm-thick SiO_2_ dielectric layer which isolates the Bi_2_Te_3_ film and Si substrate and avoids the interference of the substrate on the transport measurements. The target material consists of highly pure and uniform Bi (99.999%) and Te (99.999%) elements with the Bi: Te atomic ratio of 2:3. Prior to loading into the growth chamber, the substrates are firstly cleaned in acetone by ultrasonic wave for 15 minutes to remove the organic contaminations. Pre-growth annealing at 500 °C for 40 minutes is performed to remove the native oxides from the substrate. High-quality polycrystalline thin films are deposited at the optimized substrate temperature of 300 °C for 20 minutes, and the working pressure is set at 40 Pa with flowing Ar_2_ as the working gas in the rate of 50 sccm.

## Additional Information

**How to cite this article**: Yao, J. D. *et al.* Polarization dependent photocurrent in the Bi_2_Te_3_ topological insulator film for multifunctional photodetection. *Sci. Rep.* **5**, 14184; doi: 10.1038/srep14184 (2015).

## Supplementary Material

Supplementary Information

## References

[b1] Xia Y. *et al.* Observation of a large-gap topological-insulator class with a single Dirac cone on the surface. Nat Phys 5, 398–402, 10.1038/nphys1274 (2009).

[b2] Hsieh D. *et al.* Observation of Time-Reversal-Protected Single-Dirac-Cone Topological-Insulator States in Bi_2_Te_3_ and Sb_2_Te_3_. Phys Rev Lett 103, 10.1103/PhysRevLett.103.146401 (2009). 19905585

[b3] Pan Z. H. *et al.* Electronic Structure of the Topological Insulator Bi_2_Se_3_ Using Angle-Resolved Photoemission Spectroscopy: Evidence for a Nearly Full Surface Spin Polarization. Phys Rev Lett 106, 10.1103/PhysRevLett.106.257004 (2011). 21770666

[b4] Hsieh D. *et al.* A topological Dirac insulator in a quantum spin Hall phase. Nature 452, 970–974, 10.1038/nature06843 (2008). 18432240

[b5] Alpichshev Z. *et al.* STM Imaging of Electronic Waves on the Surface of Bi_2_Te_3_: Topologically Protected Surface States and Hexagonal Warping Effects. Phys Rev Lett 104, 10.1103/PhysRevLett.104.016401 (2010). 20366373

[b6] Zhang T. *et al.* Experimental Demonstration of Topological Surface States Protected by Time-Reversal Symmetry. Phys Rev Lett 103, 10.1103/PhysRevLett.103.266803 (2009). 20366330

[b7] Roushan P. *et al.* Topological surface states protected from backscattering by chiral spin texture. Nature 460, 1106–1109, 10.1038/nature08308 (2009). 19668187

[b8] Zhang X., Wang J. & Zhang S. C. Topological insulators for high-performance terahertz to infrared applications. Phys Rev B 82, 245107 (2010).

[b9] Hosur P. Circular photogalvanic effect on topological insulator surfaces: Berry-curvature-dependent response. Phys Rev B 83, 10.1103/PhysRevB.83.035309 (2011).

[b10] Fregoso B. M., Wang Y. H., Gedik N. & Galitski V. Driven electronic states at the surface of a topological insulator. Phys Rev B 88, 10.1103/PhysRevB.88.155129 (2013).

[b11] Shao J. M., Li H. & Yang G. W. Warping effect-induced optical absorbance increment of topological insulator films for THz photodetectors with high signal-to-noise ratio. Nanoscale 6, 3513–3517, 10.1039/c3nr06506e (2014). 24573493

[b12] McIver J. W., Hsieh D., Steinberg H., Jarillo-Herrero P. & Gedik N. Control over topological insulator photocurrents with light polarization. Nat Nanotechnol 7, 96–100, 10.1038/nnano.2011.214 (2012). 22138862

[b13] Wang Y. H., Steinberg H., Jarillo-Herrero P. & Gedik N. Observation of Floquet-Bloch states on the surface of a topological insulator. Science 342, 453–457, 10.1126/science.1239834 (2013). 24159040

[b14] Yan Y. *et al.* Topological surface state enhanced photothermoelectric effect in Bi_2_Se_3_ nanoribbons. Nano letters 14, 4389–4394, 10.1021/nl501276e (2014). 25046135

[b15] Olbrich P. *et al.* Room-Temperature High-Frequency Transport of Dirac Fermions in Epitaxially Grown Sb_2_Te_3_- and Bi_2_Te_3_-Based Topological Insulators. 10.1103/PhysRevLett.113.096601 (2014). 25215999

[b16] Zhang H. B., Yu H. L. & Yang G. W. Experimental evidence of the nanoscaled topological metallic surface state of Bi_2_Te_3_ and Sb_2_Te_3_ films. EPL (Europhysics Letters) 95, 56002, 10.1209/0295-5075/95/56002 (2011).

[b17] Zhang S. X. *et al.* Epitaxial thin films of topological insulator Bi_2_Te_3_ with two-dimensional weak anti-localization effect grown by pulsed laser deposition. Thin Solid Films 520, 6459–6462, 10.1016/j.tsf.2012.07.012 (2012).

[b18] Le P. H., Wu K. H., Luo C. W. & Leu J. Growth and characterization of topological insulator Bi_2_Se_3_ thin films on SrTiO_3_ using pulsed laser deposition. Thin Solid Films 534, 659–665, 10.1016/j.tsf.2013.01.104 (2013).

[b19] Yao J., Shao J., Wang Y., Zhao Z. & Yang G. Ultra-broadband and high response of the Bi_2_Te_3_–Si heterojunction and its application as a photodetector at room temperature in harsh working environments. Nanoscale 7, 12535–12541, 10.1039/C5NR02953H (2015). 26138000

[b20] Zhang H. B. *et al.* Magnetoresistance switch effect of a Sn-doped Bi_2_Te_3_ topological insulator. Adv Mater 24, 132–136, 10.1002/adma.201103530 (2012). 22135051

[b21] Veldhorst M. *et al.* Josephson supercurrent through a topological insulator surface state. Nat Mater 11, 417–421, 10.1038/nmat3255 (2012). 22344327

[b22] Zhang S. *et al.* Magneto-resistance up to 60 Tesla in topological insulator Bi_2_Te_3_ thin films. Appl Phys Lett 101, 202403 (2012).

[b23] Peng H., Xie C., Schoen D. T. & Cui Y. Large Anisotropy of Electrical Properties in Layer-Structured In2Se3 Nanowires. Nano letters 8, 1511–1516, 10.1021/nl080524d (2008). 18407699

[b24] Xue D. J. *et al.* Anisotropic photoresponse properties of single micrometer-sized GeSe nanosheet. Adv Mater 24, 4528–4533, 10.1002/adma.201201855 (2012). 22806941

[b25] Ils P. *et al.* Linear polarization of photoluminescence emission and absorption in quantum-well wire structures: Experiment and theory. Phys Rev B 51, 4272–4277, 10.1103/PhysRevB.51.4272 (1995). 9979268

[b26] Akiyama H., Someya T. & Sakaki H. Optical anisotropy in 5-nm-scale T-shaped quantum wires fabricated by the cleaved-edge overgrowth method. Phys Rev B 53, R4229–R4232 (1996). 10.1103/physrevb.53.r4229 9984076

[b27] Vouilloz F. *et al.* Effect of lateral confinement on valence-band mixing and polarization anisotropy in quantum wires. Phys Rev B 57, 12378–12387 (1998).

[b28] Wang J., Gudiksen M. S., Duan X., Cui Y. & Lieber C. M. Highly polarized photoluminescence and photodetection from single indium phosphide nanowires. Science 293, 1455–1457, 10.1126/science.1062340 (2001). 11520977

[b29] Trushin M. & Schliemann J. Anisotropic photoconductivity in graphene. EPL (Europhysics Letters) 96, 37006, 10.1209/0295-5075/96/37006 (2011).

[b30] Kim M. *et al.* Polarization dependence of photocurrent in a metal-graphene-metal device. Appl Phys Lett 101, 073103, 10.1063/1.4745787 (2012).

[b31] Zhang H. B. *et al.* Weak localization bulk state in a topological insulator Bi_2_Te_3_ film. Phys Rev B 86, 075102 (2012).

