# Supplementary material for: Polarization dependent photocurrent in the Bi2Te3 topological insulator film for multifunctional photodetection
Source: Sci Rep. 2015 Sep 16;5:14184. doi: 10.1038/srep14184 (PMC4570977; doi:10.1038/srep14184)
Supplement: Supplementary Information [file srep14184-s1.doc]

**Supplementary Information**

**Polarization dependent photocurrent in the Bi_2_Te_3_ topological insulator film for multifunctional photodetection**

J. D. Yao, J. M. Shao, S. W. Li, D. H. Bao, G. W. Yang

**Supplementary Section S1**

The current-voltage (I-V) characteristic of the Bi_2_Te_3_ photodetector is shown in Fig. S1. The relatively good linearity of the line reveals good ohmic contact between the Pt electrodes and the Bi_2_Te_3_ film.

**Figure S1**. The I-V curve of Bi_2_Te_3_ with Pt electrodes.

**Supplementary Section S2**

**Experimental setup configuration.** Fig. S2(a) illustrates the detail experiment measurement setup in our experiment. The output side of the 635 nm laser was fixed with a laser holder and illuminated globally on the sample. To obtain an isotropic light source, firstly, a polarizer and l/4 waveplate kept at 45 degrees were used to generate the circularly polarized light. Then, the linear polarization light was generated with another polarizer. Lights with different polarization directions are obtain by rotating the polarizer. The extinction ratio of the polarizer used in this experiment is better than 10^5^. Angle dependent power of the polarized light was measure and the result is shown in Fig. S2(b). The power is relatively isotropy, with little fluctuation origin from the unavoidable instrument as well as operating errors. The spot size of the laser light is around 3 cm in diameter, which is sufficient to cover the whole device and rule out the thermoelectric effects due to inhomogeneous heating. The photocurrent measurements were performed in a two pole configuration and the data was collected by a Keithley 4200-SCS semiconductor parameter analyzer. All measurements were performed at room temperature under ambient condition.

(b)

(a)


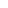


4200 SCS


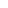


Substrate

Probe

Bi_2_Te_3_ film

Probe Station


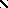


Laser

POL

LH

POL

1/4 Waveplate

**Figure S2**. (a) Schematic diagram of the experiment measurement setup. LH: Laser Holder, POL: Linear Polarizer, SCS: Semiconductor Parameter Analyzer. (b) Angle dependent power of the polarized light.

**Supplementary Section S3**

**Polarization dependent photocurrent of the sample rotated by 90°.** The photocurrent response of the TI Bi_2_Te_3_ film to 635 nm linearly polarized light after it is rotated by 90° is show in Fig. S3. The inset in (a) is schematic for the photocurrent transport measurement and the definition of the parameters keeps the same, where **A** is the light’s vector potential, indicating its polarization direction, **E**_0_ is the dc electric field caused by the bias voltage, and *θ* is the angle between them. Compared to the data in Fig. 2, the main axis of the fit in the polar diagram is offset by about 10 degree from the zero-axis and the $\mathbf{J}_{ph}^{\max}/\mathbf{J}_{ph}^{\min}$ ratio of the rotated sample equal 2 while the unrotated one exhibits a ration of 1.6. This may be due to some unintentional changes caused by the rotated process and the randomness during the measurement. Though there are some subtle differences, the general trends almost keep the same. That is, the photocurrent value is the maximum when the polarization of the light is parallel to the direction of the bias voltage and reaches a minimum value when the two are perpendicular. Therefore, it can be unambiguously claimed that we have realized the experimental demonstration of the anisotropic photocurrent of the TI Bi_2_Te_3_ film.

**Figure S3**. Polarization dependent photocurrent of the TI Bi_2_Te_3_ film (a) in Cartesian coordinate, and (b) in polar coordinates. The black dots are the experimental data and the red lines are the sinusoidal function fittings.
